# Comprehensive Analysis of Physicochemical Properties and Sensory Attributes of Original-Cut Potato Chips in the Chinese Market

**DOI:** 10.3390/foods13244158

**Published:** 2024-12-22

**Authors:** Guangcan Cui, Ting Wang, Zeyu Cai, Jianglin Liu, Yutong Hu, Qingguo Wang, Tengfei Liu

**Affiliations:** 1 College of Food Science and Engineering, Shandong Agricultural University, Tai’an 271018, China; cgc898t@163.com (G.C.); 18532096582@163.com (Z.C.); liujianglin3825@163.com (J.L.); huyutong8067@163.com (Y.H.); wqgyyy@126.com (Q.W.); 2 Tai’an Academy of Agricultural Sciences, Tai’an 271000, China; sdau0521@126.com

**Keywords:** potato chips, physicochemical properties, sensory attributes, acrylamide, texture, color, Chinese market

## Abstract

This study investigates the Chinese market’s physicochemical properties and sensory attributes of 14 original-cut potato chip brands. Color characteristics, compositional analysis, sugar content, acrylamide levels, and textural properties were examined alongside sensory evaluations. Significant variations were observed across all the parameters. Color analysis revealed diverse L*, a*, and b* values, with total color difference (ΔE) strongly correlating with sensory scores (r = 0.73, *p* < 0.01). A compositional analysis showed substantial differences in protein (5.19–8.51%), fat (27.91–40.16%), and moisture (0.67–3.78%) contents. Acrylamide levels varied widely (166.7–1101.78 mg/kg), positively correlating with the sucrose content (r = 0.57, *p* < 0.05). A textural analysis demonstrated significant variations in hardness (379.38–1103.6 gf) and fracturability (167.5–857.77 gf), with fracturability negatively correlating with sensory scores (r = −0.75, *p* < 0.01). A sensory evaluation revealed distinct brand preferences, with the total scores ranging from 65 to 85. This comprehensive analysis provides valuable insights into the complex interplay between the physicochemical properties and consumer perception of potato chips in the Chinese market and offers potential directions for product optimization and quality control in the snack food industry, inspiring hope and innovation among industry professionals.

## 1. Introduction

Potato chips are among the most widely consumed snack foods globally, cherished for their distinctive crunch, flavor, and convenience. The global potato chips market has witnessed substantial growth, with a valuation exceeding USD 30 billion in recent years, driven by factors such as urbanization, changing lifestyles, and an increasing preference for ready-to-eat foods (https://www.mordorintelligence.com/industry-reports/potato-chips-market [accessed on 1 December 2024]). However, the industry faces significant challenges in meeting consumer demands for healthier options without compromising taste and quality.

Sensory attributes—taste, texture, aroma, and appearance—are critical determinants of consumer acceptance and preference for potato chips [1]. Color and appearance are paramount among these attributes, as they are the first parameters consumers evaluate and can significantly influence purchase decisions even before consumption [2]. The characteristic golden-yellow color of potato chips, resulting from deep-fat frying at temperatures between 150 and 190 °C for 3–5 min, is associated with proper cooking and perceived quality [3]. Color uniformity and the absence of defects such as dark spots play a fundamental role in acceptable appearance [4].

The Maillard reaction, a complex series of non-enzymatic browning reactions between reducing sugars and amino acids, particularly asparagine, is the primary driver of color development in potato chips [5]. This reaction contributes to the desirable flavors, aromas, and colors of fried foods and leads to the formation of melanoidins. These brown nitrogen-containing polymers significantly affect the visual appeal of the product [6]. Understanding and controlling the Maillard reaction is crucial, as excessive browning can result in off-colors that negatively impact consumer acceptance [7].

However, the Maillard reaction also leads to acrylamide formation, a contaminant that has raised significant health concerns [8]. Acrylamide forms when foods are subjected to high temperatures (above 120 °C) during frying, roasting, or baking, particularly in low moisture conditions. The health implications of acrylamide are significant, as animal studies have demonstrated its genotoxic and carcinogenic potential, raising concerns about increased cancer risk across all age groups [9]. Consequently, the International Agency for Research on Cancer (IARC) has classified acrylamide as a probable human carcinogen [10,11].

In response to these health concerns, regulatory bodies have established benchmark levels for acrylamide in various food products, including potato chips. Initially, the European Commission set an indicative value of 1000 µg/kg for potato crisps [12]. However, as scientific understanding of acrylamide’s risks has evolved and mitigation strategies have improved, this benchmark has been progressively lowered. The current benchmark level stands at 750 µg/kg (https://eur-lex.europa.eu/eli/reg/2017/2158/oj [accessed on 1 December 2024]), reflecting the ongoing efforts to minimize consumer exposure to this contaminant. These regulatory measures have spurred significant research and development efforts within the food industry to reduce acrylamide formation while maintaining product quality and consumer acceptance.

A comprehensive meta-analysis of 47 articles, encompassing 230 studies, has provided valuable insights into acrylamide concentrations across various food categories [13]. This analysis, which considered factors such as product type, country of origin, and analytical methods, revealed that potato-based foods contained the highest average acrylamide levels (740.33 µg/kg), followed by fried foods (328.65 µg/kg), breakfast cereals (263.27 µg/kg), and coffee (234.54 µg/kg) [13]. These findings align with the European Food Safety Agency’s (EFSA) identification of processed potatoes, coffee, and cereal-based foods as the primary sources of dietary acrylamide exposure [14]. Notably, this meta-analysis confirmed that potato-based products, including chips and crisps, contained the highest average acrylamide levels among the food categories studied. This finding is consistent with previous research [15], which reported that acrylamide concentrations in some potato chips could reach up to 3000 μg/kg or even exceed 4000 μg/kg in extreme cases.

The high levels of acrylamide in potato chips are particularly concerning due to their popularity as a snack food, potentially leading to significant dietary exposure [16]. Identifying acrylamide as a health risk has led to increased scrutiny of potato chip production processes and a push for mitigation strategies [17]. However, the challenge lies in reducing acrylamide formation without compromising the sensory qualities consumers expect from potato chips [18]. This presents a complex problem for food scientists and manufacturers, requiring innovative approaches to balance food safety with product quality and consumer acceptance. This need for innovation should not be seen as a hurdle but as a motivating factor that drives the industry towards better and safer products.

In response to growing health awareness, consumers increasingly seek snacks with improved nutritional profiles, such as reduced fat content and enhanced protein levels [19]. Traditional deep-fat frying methods lead to high oil absorption in potato chips, with the oil content ranging from 35% to 45% by weight. This contributes to high caloric values and potential health risks such as obesity and cardiovascular diseases [20]. Therefore, reducing the oil content without adversely affecting sensory qualities has become a focal point in potato chip production [21].

Understanding the physicochemical properties of potato chips is essential for optimizing product quality. Protein content can influence the Maillard reaction, potentially affecting color and flavor development [22]. Fat content contributes to mouthfeel and flavor but must be balanced to avoid adverse health perceptions [23]. Moisture content affects texture, with lower moisture levels generally contributing to increased crispness, a highly valued attribute in potato chips [24]. The pH of potato slices can influence enzymatic reactions and acrylamide formation during frying [17]. Controlling these variables is crucial for producing potato chips that meet consumer expectations while addressing health concerns.

China, as the world’s largest potato producer, has experienced rapid growth in its potato processing industry [25]. However, previous research in this context has been predominantly limited to investigating individual aspects of commercially available potato chips [15,26] without comprehensively integrating these factors with consumer preferences and sensory evaluation. This fragmented approach has left a significant gap in understanding the complex interplay between physicochemical properties, sensory attributes, and consumer acceptance in the Chinese market.

The burgeoning consumer demand for healthier snack options necessitates a more holistic approach that simultaneously addresses nutritional improvements and sensory appeal. This is particularly relevant in the Chinese context, where increasing health consciousness among consumers is reshaping snack food preferences. Furthermore, the substantial economic significance of the potato chip industry in China demands continuous innovation to maintain market share while addressing emerging health concerns [25].

Given these considerations, research is needed to bridge the gap between product characteristics and consumer preferences in the Chinese potato chip market. This study aimed to investigate the relationships between the sensory attributes and physicochemical properties of potato chips in the Chinese market. The objectives were to (1) characterize the nutritional composition, texture properties, and acrylamide content of 14 commercial potato chip brands and (2) evaluate how these physicochemical characteristics correlate with consumer sensory preferences. The findings will provide valuable insights for manufacturers to optimize product quality while meeting consumer expectations and health requirements.

## 2. Materials and Methods

### 2.1. Food Materials

Commercial potato chip samples were collected during December 2023 from diverse retail outlets, including major supermarkets, local convenience stores, and e-commerce platforms in Tai’an City, Shandong Province, China. Five bags were randomly purchased from each brand, and about 15 g of samples were randomly taken from three of these bags for subsequent analysis. The samples were stored in their original packaging at room temperature (approximately 25 °C) until testing. For each sample, chips of similar shape and size were selected. Measurements were taken immediately after opening the packaging.

### 2.2. Chemicals and Reagents

Methanol (HPLC grade) and acetonitrile (HPLC grade) were purchased from Tianjin Yongda Chemical Reagent Co., Ltd. (Tianjin, China). n-Hexane (chromatographic grade), anhydrous magnesium sulfate (analytical grade, ≥99.0%), sodium chloride (analytical grade, ≥99.5%), aluminum oxide (analytical grade, ≥99.5%), sucrose (analytical grade, ≥99.0%), glucose (analytical grade, ≥98.0%), and fructose (analytical grade, ≥99.5%) were obtained from Tianjin Kaitong Chemical Reagent Co., Ltd. (Tianjin, China). Acrylamide (purity ≥ 99%) was purchased from Shanghai Macklin Biochemical Technology Co., Ltd. (Shanghai, China).

### 2.3. Color Characteristics of Potato Chips

The color characteristics of ground potato chip samples were measured using a colorimeter (CR-400, Minolta Co., Osaka, Japan) calibrated with a standard white plate (L* = 97.06, a* = 0.04, b* = 2.01). In this system, L* represents lightness (range 0–100; 0 = pure black, 100 = pure white), a* represents the red–green axis (range −128 to +127; −128 = green, +127 = red), and b* represents the yellow–blue axis (range −128 to +127; −128 = blue, +127 = yellow). To obtain a uniform sample color, the potato chips were ground and passed through a 40-mesh sieve, and the entire process was completed in a laboratory setting to minimize time. The ground samples were spread evenly and compacted in Petri dishes for color analysis. Each sample was measured five times. The average values of L*, a*, and b* were calculated, and the color difference ΔE was determined using the following equation:

(1) $$\Delta E=\sqrt{\left({\left(L_{0}^{*}-L^{*}\right)}^{2}+{{(a}_{0}^{*}-a^{*})}^{2}+{{(b}_{0}^{*}-b^{*})}^{2}\right)}$$

### 2.4. Quantification of Protein and Fat Content

For protein content determination, standard solutions were prepared: 0.10 mol/L HCl, 40% (*w*/*v*) NaOH, and 2% (*w*/*v*) boric acid. A mixed indicator solution of methyl red and bromocresol green was prepared according to standard protocols. Additional reagents, including potassium permanganate and sulfuric acid solutions, were prepared as needed. Approximately 0.3 g of each dried sample was weighed into digestion tubes. To each tube, 10 mL of concentrated H_2_SO_4_ was added. The samples were digested in a graphite digestion apparatus (SH420, Hanon Advanced Technology Group, Jinan, China) using a programmed temperature profile: 100 °C for 10 min, 280 °C for 10 min, and 400 °C for 40 min. Following initial digestion, 1 mL of H_2_O_2_ was added, and the samples were further digested at 200 °C for 10 min, 320 °C for 30 min, and 400 °C for 30 min. A final addition of 0.5 mL H_2_O_2_ was made, followed by digestion at 200 °C for 10 min, 320 °C for 30 min, and 400 °C for 60 min, or until solutions became apparent. Total nitrogen was determined using a Kjeldahl apparatus (K1160, Hanon Advanced Technology Group, Jinan, China). The system was calibrated, and blank runs were performed before sample analysis. For each sample, appropriate parameters were set, including sample weight, acid concentration, blank value, and protein conversion factor (6.25) [27]. Automated distillation and titration were conducted according to the manufacturer’s instructions.

Protein content was calculated using the following equation:

Protein (%) = (V2 − V1) × c(HCl) × 0.014 × 6.25 × 100%/m(2)

where V2 is the volume of HCl used for sample titration (mL), V1 is the volume of HCl used for blank titration (mL), c(HCl) is the concentration of standard HCl solution (mol/L), and m is the sample mass (g).

For fat content determination, the samples were dried at 105 °C to constant weight and ground to pass through a 40-mesh sieve. Extraction thimbles were prepared using filter paper. The dried samples (1–2 g) were accurately weighed into the thimbles. Fat extraction used a Soxhlet apparatus with petroleum ether as the solvent. The extraction process was conducted for 300 min at 60–68 °C. Following the extraction, the samples were dried and reweighed.

Fat content was calculated using the following equation:

Fat content (%) = (W2 − W3)/(W2 − W1) × 100(3)

W1 is the weight of the empty thimble, W2 is the weight of the thimble plus the sample before extraction, and W3 is the weight of the thimble plus the sample after extraction. All the analyses were performed in triplicate, and the results are expressed as mean percentage (*w*/*w*) ± standard deviation on a dry weight basis.

### 2.5. Determination of Moisture Content, pH Value, and Total Titratable Acidity (TTA)

For moisture content determination, a 10 g sample of ground potato chips was weighed and placed in a sealed weighing bottle. The weighing bottle was transferred to a drying oven, uncapped, and maintained at a temperature between 95 °C and 105 °C for 2–4 h. After the initial drying period, the heating was stopped, and the weighing bottle was allowed to cool before weighing. The sample was then subjected to additional 1 h drying cycles followed by weighing until the difference between consecutive weighings was less than 2 mg, at which point the sample was considered to be absolutely dry. The moisture content was calculated using the following parameters:

(4) $$Moisture\left(\%\right)=\frac{W1-W2}{W1-W3}\times 100\%$$

W1 = Weight of sample and weighing bottle before drying (g);

W2 = Weight of sample and weighing bottle after drying (g);

W3 = Weight of empty weighing bottle (g).

For pH value determination, a 10 g sample of ground potato chips was dispersed in 90 mL of distilled water in a beaker and homogenized using a homogenizer (T10 basic, IKA, Staufen, Germany). The suspension was allowed to stand at room temperature for 30 min. The pH value of the supernatant was measured using a pH meter (S220, METTLER TOLEDO, Greifensee, Switzerland).

The total titratable acidity was determined by dispersing 10 g of ground potato chip sample in 90 mL of distilled water in a beaker, followed by homogenization using a homogenizer (T10 basic, IKA, Staufen, Germany). Each sample was mixed with 0.5 mL of 1.0% phenolphthalein indicator and titrated with 0.1 M NaOH. The endpoint was determined when a pink color persisted for 30 s. The titratable acidity was calculated using the following equation:

(5) $$TTA\%=\frac{c\times (V_{3}-V_{2})\times K}{m}\times \frac{V_{0}}{V_{1}}\times 100\%$$

where

c = Concentration of standard NaOH solution (mol/L);

V_3_ = Volume of NaOH consumed by sample (mL);

V_2_ = Volume of NaOH consumed in blank titration (mL) m = Sample mass (g);

V_0_ = Total volume of diluted sample (mL);

V_1_ = Volume of sample aliquot taken for titration (mL);

K = Conversion factor for the predominant acid (0.090 for lactic acid).

### 2.6. Quantification of Sugar and Acrylamide Content

The glucose, fructose, and sucrose contents in the potato chip samples were analyzed based on the method of our previous report [28] with slight modifications. Weigh 50 mg of the ground–dried potato chips sample and add 1 mL of 70% (*v*/*v*) methanol. Mix thoroughly and extract at 4 °C for 12 h—centrifuge at 12,100× *g* for 2 min (Z 216 MK, HERMLE Labortechnik GmbH, Wehingen, Germany). Filter the supernatant through a 0.22 μm organic filter using a syringe. Accurately pipette 200 μL of the filtered sample into an LC vial for mass spectrometry analysis. In brief, the sugar determination employed a Vanquish UHPLC system coupled with a Q Exactive Plus Mass Spectrometer (Thermo Fisher Scientific, Bremen, Germany) for precise sugar analysis. Our separation was achieved using an Accucore 150 Amide HILIC column operated at 70 °C with an isocratic mobile phase (84:16 acetonitrile–water containing 10 mM ammonium formate) at a 0.8 mL/min flow rate. For detection, we utilized Selected Ion Monitoring (SIM) in the negative ESI mode, monitoring specific masses (180 and 342 *m*/*z*) with optimized parameters (3 *m*/*z* isolation window, 1 × 10^6^ AGC target value, 35 k resolution). We also performed MS2 analysis using SIM + PRM modes for the structural confirmation of glucose, fructose (*m*/*z* 179), and sucrose (*m*/*z* 341), ensuring accurate sugar identification and quantification. All the samples were analyzed in triplicate, and the results are expressed as mean ± standard deviation in mg/kg of sample.

For acrylamide analysis, 1.0 g of the ground–dried potato chip samples were accurately weighed. Acrylamide extraction and quantification were performed following the method described in our previous report [29].

### 2.7. Texture Analysis

The texture parameters of the potato chips were measured using a texture analyzer (TA.XTC-20, Shanghai Baosheng Industrial Development Co., Ltd., Shanghai, China). The samples were placed directly under the probe, and a TA/0.25S spherical investigation was used for puncture tests. The sample orientation was consistent for each test. Parameters were set as follows: pre-test speed 1.0 mm/s, test speed 0.5 mm/s, post-test speed 0.5 mm/s, distance 1.5 mm, trigger force 5 g. The distance corresponding to the first peak represents the crispness of the potato chip, and the average force from the first peak to 1 s represents the hardness. All the texture measurements were performed in three replicates for each brand, with samples taken from different packets to ensure representativeness.

### 2.8. Sensory Evaluation

The sensory attributes of the potato chip samples were determined according to ISO 11136:2014 [30]. A panel of 30 members was selected from the university campus and trained before the sensory tests to assess the characteristics of the potato chips in this study. The potato chip samples were placed on 14 disposable white plates, each containing three intact chips of similar appearance, randomly labeled with three-digit codes. The panelists evaluated the samples by tasting, observing, and smelling, scoring them based on five criteria: taste, texture, appearance, color, and odor, each rated on a three-level scale [31]. A specific interval was maintained between evaluations of each sample, with the panelists rinsing their mouths with water and avoiding discussions. The sensory evaluation standards for fried potato chips are shown in Table 1.

### 2.9. Data Analysis

Experimental data were plotted using GraphPad Prism 8.3.0 and analyzed using IBM SPSS Statistics 26. Image processing and output were performed with Adobe Illustrator. All the experiments were designed as wholly randomized, each repeated three times. Data are expressed as mean ± standard deviation. An analysis of variance (ANOVA) was used for difference analysis, with mean separation and least significant difference (LSD) tests. Differences were considered statistically significant at *p* < 0.05.

## 3. Results and Discussion

### 3.1. Color Analysis of 14 Original-Cut Potato Chip Brands in the Chinese Market

**Figure 1:**
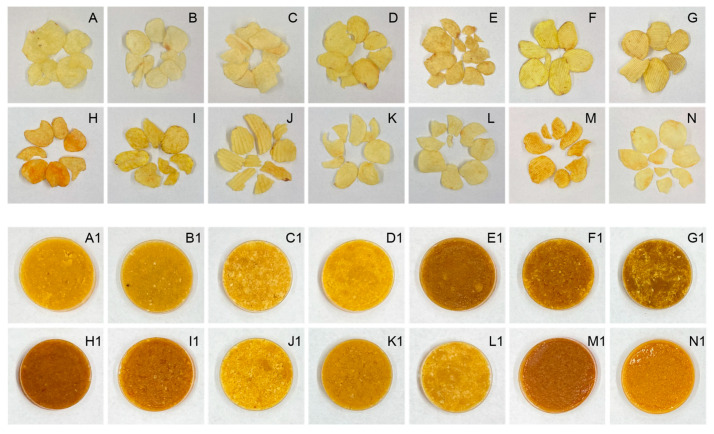
Visual representation of 14 original-cut potato chip brands (**A**–**N**) in the Chinese market. **Top panel**: Individual potato chip samples. **Bottom panel**: Crushed samples for color measurement.

We systematically sampled commercially available brands to comprehensively evaluate the original-cut potato chip products in the Chinese market. Fourteen distinct brands of original-cut potato chips were procured from various retail outlets (Figure 1, Table S1). The selection process aimed to encompass various domestic and international manufacturers to ensure a representative Chinese potato chip market sample.

Color is a critical quality parameter for fried potato products, significantly influencing consumer perception and purchase decisions [26]. The color characteristics of the 14 original-cut potato chip brands were analyzed using the CIE Lab* color space and ΔE values (Table 2). Significant variations in the color parameters were observed across the samples (*p* < 0.05). The L* values, indicating lightness, ranged from 41.75 ± 0.44 (Brand M) to 61.38 ± 0.99 (Brand C), with Brands C, D, J, and L exhibiting the highest lightness (*p* < 0.05). The a* values, representing redness, varied from 2.66 ± 0.14 (Brand B) to 10.26 ± 0.18 (Brand H), with Brands H and M showing significantly higher redness (*p* < 0.05). The b* values, indicating yellowness, ranged from 25.07 ± 0.34 (Brand M) to 43.07 ± 0.48 (Brand D), with Brand D demonstrating the highest yellowness (*p* < 0.05). The overall color difference (ΔE) values ranged from 49.69 ± 0.45 (Brand M) to 74.35 ± 0.95 (Brand D), with Brand D exhibiting the most significant color difference from the standard white plate (*p* < 0.05). This color development not only affects the product’s visual appeal but also indicates flavor development and overall quality to consumers [26]. These results highlight the substantial color diversity among the potato chip brands in the Chinese market, which may influence consumer perception and preference.

### 3.2. Nutritional and Chemical Properties of 14 Original-Cut Potato Chip Brands

With the development of society, consumers are increasingly paying attention to the nutritional components of snack foods [32]. This growing health consciousness has prompted a need for a comprehensive understanding of the nutritional and physicochemical properties of potato chips. The compositional analysis revealed significant variations in protein content, fat content, moisture, pH, and total titratable acidity (TTA) among the samples (Table 3). The protein content ranged from 5.19 ± 0.12% (Brand B) to 8.51 ± 0.05% (Brand I), with Brand I exhibiting significantly higher protein content than all the other brands (*p* < 0.05). The fat content varied considerably, from 27.91 ± 1.11% (Brand D) to 40.16 ± 1.82% (Brand N), with Brands M and N showing significantly higher fat content (*p* < 0.05). The moisture content ranged from 0.67 ± 0.07% (Brand M) to 3.78 ± 0.05% (Brand L), indicating substantial differences in chip crispness. The pH values of the samples were generally slightly acidic, ranging from 5.96 ± 0.01 (Brand J) to 6.29 ± 0.04 (Brand A), with Brands A and D exhibiting significantly higher pH values (*p* < 0.05). The total titratable acidity (TTA) varied from 0.86 ± 0.08% (Brand B) to 1.46 ± 0.11% (Brand I), with Brand I showing significantly higher TTA compared to most other brands (*p* < 0.05). These results highlight the considerable diversity in the compositional characteristics of potato chips in the Chinese market, which may influence their sensory properties, shelf life, and nutritional value. The higher protein content in some brands, particularly Brand I, may be due to protein-rich potato varieties or the addition of protein-based flavor enhancers (Table S2). The wide range of fat content reflects different frying techniques and oil absorption rates, significantly impacting sensory attributes and nutritional profiles.

### 3.3. Sugar Content and Acrylamide Levels in 14 Original-Cut Potato Chip Brands

The analysis of the sugar content and acrylamide levels in the 14 original-cut potato chip brands from the Chinese market revealed significant variations across the samples (Table 4). The glucose content ranged from undetectable levels in several brands (A, C, D, E, I, J, and M) to 17.56 ± 0.8 mg/g in Brand F, which was significantly higher than all the other brands (*p* < 0.05). This elevated glucose content was attributed to adding glucose as a flavoring agent, as disclosed in Brand F’s ingredient list. The fructose content varied from 0.78 ± 0.02 mg/g (Brand C) to 6.9 ± 1.03 mg/g (Brand D), with Brand D showing significantly higher levels (*p* < 0.05). The sucrose content ranged from 1.28 ± 0.15 mg/g (Brand C) to 8.78 ± 0.79 mg/g (Brand G), with Brand G exhibiting significantly higher sucrose levels than all the other brands (*p* < 0.05).

Notably, the acrylamide levels showed substantial variation, ranging from 166.7 ± 9.6 μg/kg (Brand L) to 1101.78 ± 22.55 μg/kg (Brand M). The mean acrylamide content (430.39 ± 283.29 μg/kg) observed in the 14 potato chip products aligns with previously reported levels (564 ± 285 μg/kg) for potato products in the Chinese market [15]. This consistency suggests a degree of stability in the acrylamide levels within the Chinese potato product industry over time. However, it is essential to note that the current study focuses specifically on potato chips, while the previous data encompassed a broader range of potato products, which may explain the differences between the acrylamide levels observed in this study and the broader range of values reported previously.

Brand M had significantly higher acrylamide levels compared to all the other brands, followed by Brand G (908.75 ± 53.5 μg/kg) and Brand N (671.73 ± 23 μg/kg) (*p* < 0.05). Notably, Brand M also had the highest sucrose content (8.78 ± 0.79 mg/g) compared to the other brands, including Brand N (6.27 ± 0.33 mg/g), which suggests a potential correlation between higher sucrose content. These results highlight the considerable differences in sugar composition and acrylamide formation among potato chip brands in the Chinese market, which may affect flavor profiles, consumer acceptance, and potential health concerns related to acrylamide exposure.

The substantial variations in acrylamide levels across the brands underscore the challenges in controlling acrylamide formation during potato chip production. Some brands’ exceptionally high acrylamide levels, particularly Brand M (1101.78 ± 22.55 μg/kg), raise significant concerns about potential health risks. These levels far exceed the benchmark levels set by the European Commission (750 μg/kg for potato crisps) [33], highlighting the urgent need for improved processing techniques or raw material selection to mitigate acrylamide formation without compromising sensory qualities.

### 3.4. Textural Properties of 14 Original-Cut Potato Chip Brands

The textural properties were evaluated by measuring the hardness and fracturability (Table 5). Significant parameter variations were observed across the samples (*p* < 0.05). the hardness values ranged from 379.38 ± 20.92 gf (Brand J) to 1103.6 ± 22.03 gf (Brand E), with Brand E exhibiting significantly higher hardness compared to all the other brands (*p* < 0.05). Brand F also showed notably high hardness (1016.01 ± 50.14 gf), substantially different from most other brands. The fracturability measurements varied from 167.5 ± 24.74 gf (Brand A) to 857.77 ± 58.46 gf (Brand E), with Brand E again demonstrating significantly higher fracturability than all the other brands (*p* < 0.05). Interestingly, some brands showed similar values for both hardness and fracturability (e.g., Brand I: 660.88 ± 59.47 gf for both parameters; Brand M: 724.62 ± 16.6 gf for both), while the others displayed marked differences between these two textural attributes. These results highlight the diverse textural characteristics among potato chip brands in the Chinese market, which may significantly influence consumer perception and preference for different products.

The strong negative correlation between the fracturability and sensory scores (r = −0.75, *p* < 0.01) suggests that Chinese consumers may prefer potato chips with a less brittle texture. This preference could be related to cultural eating habits or expectations of freshness associated with a slightly less crisp texture. The significant negative correlation between moisture content and hardness (r = −0.63, *p* < 0.05) underscores the critical role of moisture control in achieving the desired textural attributes.

### 3.5. Sensory Evaluation of 14 Original-Cut Potato Chip Brands in the Chinese Market

**Figure 2:**
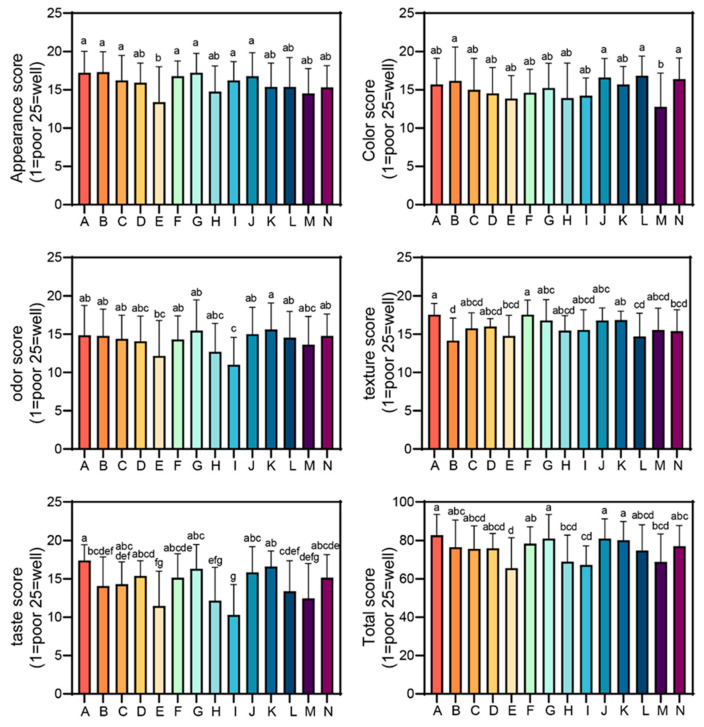
Sensory evaluation scores for 14 original-cut potato chip brands (A–N) available in the Chinese market. The scores are presented for appearance, color, odor, texture, taste, and total sensory evaluation on a 25-point scale (1 = poor, 25 = excellent). All the values are indicated as mean ± SD (*n* = 30). Different letters above bars indicate significant differences (*p* < 0.05) between the brands for each attribute.

The sensory evaluation revealed significant variations across multiple attributes for the 14 potato chip brands (A through N) (Figure 2). The appearance scores ranged from approximately 13 to 18, with Brands A, B, G, and J receiving among the highest ratings. The color scores showed less variation, ranging from about 13 to 17, with Brands A, J, L, and N scoring slightly higher than others. The odor evaluations demonstrated more variability, with scores ranging from 11 to 16. Brand K received the highest odor rating. The texture scores varied from approximately 14 to 18, with Brands A and F scoring significantly higher than most others. The taste evaluations showed the most pronounced differences, with the scores ranging from 10 to 17. Brand A outperformed all the others in taste. The total sensory scores, combining all the attributes, ranged from about 65 to 83, with Brands A, G, J, and K achieving the highest overall ratings. These results highlight the diverse sensory profiles among potato chip brands, with certain brands consistently outperforming others across multiple attributes. Brand A excelled in taste and overall sensory appeal, outperforming others in most categories. The wide range in taste scores indicates that flavor remains a crucial differentiator in the market.

The data suggests that while some brands perform well across multiple sensory aspects, others may have strengths in specific areas. This information could be valuable for manufacturers in identifying improvement areas and for consumers to make informed choices based on their sensory preferences.

### 3.6. Correlation Analysis of Physicochemical Properties and Sensory Scores of Original-Cut Potato Chips in the Chinese Market

**Figure 3:**
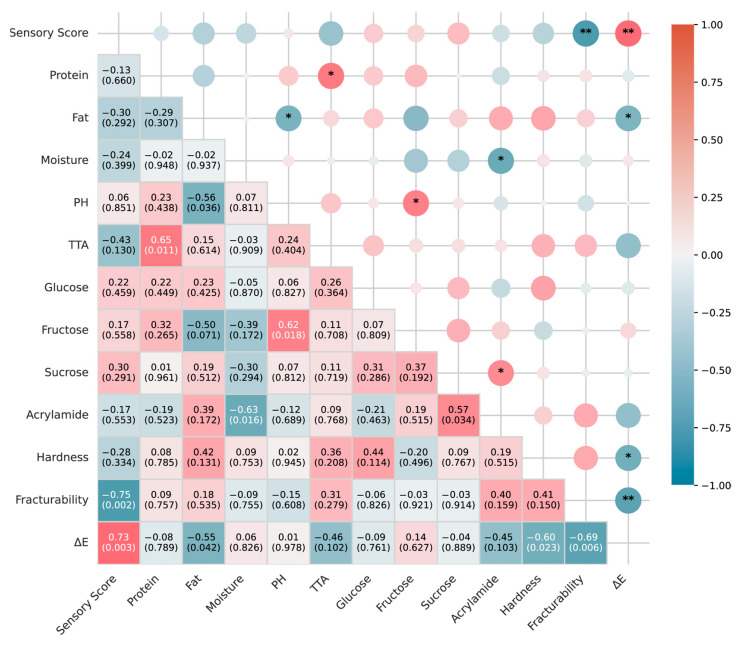
Correlation matrix heatmap of sensory scores and physicochemical properties of 14 original-cut potato chip brands in the Chinese market. The color intensity and size of the circles represent the strength of the correlation. Positive correlations are displayed in red, and negative correlations in blue. The numbers in each cell represent the correlation coefficient and its corresponding *p*-value in parentheses. Asterisks indicate statistical significance: * *p* < 0.05; ** *p* < 0.01.

The correlation analysis provided several significant relationships (Figure 3). Notably, the sensory score showed a strong positive correlation with ΔE (r = 0.73, *p* < 0.01), indicating that color difference plays a crucial role in consumer perception [34]. Conversely, a significant negative correlation was observed between the sensory score and fracturability (r = −0.75, *p* < 0.01), suggesting that less brittle chips are generally preferred. The fat content exhibited a moderate negative correlation with pH (r = −0.56, *p* < 0.05), which may influence the overall flavor profile. The acrylamide content was positively correlated with the sucrose levels (r = 0.57, *p* < 0.05). This is unsurprising, as reducing sugars may be consumed during processing, while sucrose is more stable. The sucrose content can, to some extent, reflect the total sugar content (including reducing sugars) in the raw potatoes. The reducing sugar content in raw potatoes plays a crucial role in the acrylamide content of processed products [28]. Based on our analysis, Brands A, F, and L achieved significantly lower acrylamide levels while maintaining high sensory scores (*p* < 0.05). The reduced acrylamide formation in these brands correlated with their lower sucrose content (r = 0.57, *p* < 0.05). These findings suggest that commercial-scale acrylamide reduction can be achieved through selecting raw materials with controlled sugar content without compromising product quality.

Furthermore, the moisture content showed a significant negative correlation with hardness (r = −0.63, *p* < 0.05), underscoring the impact of water content on texture properties. The correlation analysis provides valuable insights into the complex relationships between the physicochemical properties and sensory perception of potato chips in the Chinese market. The strong positive correlation between the ΔE and sensory scores reinforces the importance of visual appeal in consumer acceptance. The negative correlation between the fracturability and sensory scores challenges the conventional wisdom that higher crispness is always preferred, suggesting a more nuanced consumer preference in the Chinese market. These findings underscore the need for a holistic approach to product development that considers the interplay between various physicochemical attributes and their combined impact on sensory perception.

## 4. Conclusions

This comprehensive study of 14 original-cut potato chip brands in the Chinese market reveals significant variations in physicochemical properties and sensory attributes, providing valuable insights for the snack food industry. The strong correlations between the color parameters, textural properties, and sensory scores highlight the complex interplay of the factors influencing consumer perception and preference.

Color difference (ΔE) strongly correlates with sensory scores, emphasizing the importance of visual appeal in consumer acceptance. Textural properties, particularly fracturability, play a crucial role in sensory perception, with Chinese consumers showing a preference for less brittle chips. Acrylamide levels vary widely across brands and correlate positively with sucrose content, underscoring the need for improved processing techniques to mitigate acrylamide formation without compromising sensory qualities. Compositional analysis reveals diverse approaches to potato chip production, with significant variations in protein, fat, and moisture contents influencing both nutritional profiles and sensory attributes.

These results provide a foundation for targeted product optimization strategies in the Chinese potato chip market. Manufacturers should focus on balancing color development, texture optimization, and acrylamide mitigation to meet consumer preferences while addressing health concerns. Future research could explore innovative processing techniques or raw material selection methods to achieve this balance more effectively.

Additionally, the observed preferences for specific sensory attributes in the Chinese market highlight the importance of culturally tailored product development approaches. Understanding and leveraging these market-specific preferences will be crucial for success in the competitive Chinese potato chip market as the snack food industry evolves.

## Figures and Tables

**Table 1:** Sensory evaluation criteria for fried potato chips.

Attribute	Evaluation Standard	Grade	Score
Appearance	Complete slices with minimal breakage	Good	15–20
	Fairly complete slices with some breakage	Fair	10–15
	Incomplete slices with significant breakage	Poor	5–10
Color	Uniform color without burnt spots	Good	15–20
	Relatively uniform color with occasional burnt spots	Fair	10–15
	Uneven color with many burnt spots	Poor	5–10
Odor	Strong fried potato aroma without off-flavors	Good	15–20
	Slight fried potato aroma without off-flavors	Fair	10–15
	No fried potato aroma or presence of off-flavors	Poor	5–10
Texture	Crisp with fried taste	Good	15–20
	Fairly crisp with some soft areas	Fair	10–15
	Soft without fried taste	Poor	5–10
Taste	Strong potato flavor without off-flavors	Good	15–20
	Slight potato flavor without off-flavors	Fair	10–15
	No potato flavor or presence of off-flavors	Poor	5–10

**Table 2:** Color parameters (L*, a*, and b*) and total color difference (ΔE) of 14 original-cut potato chip brands in the Chinese market.

Brand	L* Value	a* Value	b* Value	ΔE Value
A	56.37 ± 0.39 ^c^	2.91 ± 0.64 ^gh^	36.59 ± 0.95 ^cd^	67.27 ± 0.67 ^d^
B	58.58 ± 0.64 ^b^	2.66 ± 0.14 ^h^	33.46 ± 0.45 ^f^	67.51 ± 0.49 ^d^
C	61.38 ± 0.99 ^a^	3.04 ± 0.35 ^gh^	36.90 ± 0.72 ^cd^	71.69 ± 1.02 ^bc^
D	60.44 ± 0.94 ^a^	4.46 ± 0.33 ^ef^	43.07 ± 0.48 ^a^	74.35 ± 0.95 ^a^
E	45.52 ± 0.79 ^f^	5.87 ± 0.16 ^d^	27.20 ± 0.54 ^i^	53.35 ± 0.91 ^h^
F	49.96 ± 0.88 ^e^	5.96 ± 0.21 ^cd^	35.15 ± 0.60 ^e^	61.38 ± 1.00 ^f^
G	53.23 ± 2.47 ^d^	6.49 ± 0.93 ^c^	36.11 ± 1.32 ^d^	64.66 ± 2.61 ^e^
H	45.97 ± 0.56 ^f^	10.26 ± 0.18 ^a^	32.45 ± 0.65 ^g^	57.20 ± 0.82 ^g^
I	47.04 ± 1.04 ^f^	7.50 ± 0.30 ^b^	30.41 ± 0.22 ^h^	56.51 ± 0.81 ^g^
J	60.63 ± 1.90 ^a^	4.15 ± 0.99 ^f^	40.40 ± 0.49 ^b^	72.99 ± 1.51 ^ab^
K	56.30 ± 0.42 ^c^	4.86 ± 0.20 ^e^	37.50 ± 0.59 ^c^	67.82 ± 0.57 ^d^
L	60.21 ± 2.10 ^a^	3.45 ± 0.43 ^g^	36.30 ± 0.68 ^d^	70.40 ± 2.07 ^c^
M	41.75 ± 0.44 ^g^	9.89 ± 0.17 ^a^	25.07 ± 0.34 ^j^	49.69 ± 0.45 ^i^
N	52.69 ± 0.67 ^d^	7.93 ± 0.19 ^b^	34.68 ± 0.95 ^e^	63.58 ± 0.56 ^e^
Mean	53.58 ± 6.43	5.67 ± 2.54	34.66 ± 4.87	64.17 ± 7.54

Different letters indicate significant differences at *p* < 0.05. All the values are indicated as mean ± SD (*n* = 3).

**Table 3:** Compositional analysis of 14 original-cut potato chip brands.

Brand	Protein (%)	Fat (%)	Moisture (%)	pH Value	Total Titratable Acidity (TTA)%
A	6.26 ± 0.07 ^ef^	30.69 ± 1.74 ^def^	2.04 ± 0.09 ^d^	6.29 ± 0.04 ^a^	1.02 ± 0.04 ^de^
B	5.19 ± 0.12 ^h^	34.28 ± 1.30 ^bc^	2.43 ± 0.13 ^c^	6.16 ± 0.04 ^bc^	0.86 ± 0.08 ^e^
C	5.79 ± 0.17 ^g^	33.74 ± 1.59 ^bcd^	1.54 ± 0.03 ^fg^	6.05 ± 0.03 ^f^	0.89 ± 0.04 ^e^
D	7.24 ± 0.13 ^b^	27.91 ± 1.11 ^f^	1.47 ± 0.04 ^g^	6.29 ± 0.09 ^a^	1.37 ± 0.08 ^ab^
E	5.61 ± 0.05 ^g^	37.04 ± 1.06 ^ab^	3.27 ± 0.03 ^b^	6.11 ± 0.01 ^de^	1.24 ± 0.08 ^abcd^
F	7.23 ± 0.41 ^b^	36.84 ± 1.94 ^ab^	1.76 ± 0.09 ^e^	6.16 ± 0.07 ^bc^	1.40 ± 0.30 ^ab^
G	6.31 ± 0.28 ^ef^	35.02 ± 1.83 ^bc^	1.08 ± 0.06 ^h^	6.09 ± 0.02 ^e^	0.91 ± 0.03 ^e^
H	6.98 ± 0.05 ^bc^	32.52 ± 0.33 ^cde^	1.06 ± 0.07 ^h^	6.14 ± 0.08 ^bcd^	1.09 ± 0.07 ^cde^
I	8.51 ± 0.05 ^a^	34.34 ± 1.90 ^bc^	2.58 ± 0.09 ^c^	6.11 ± 0.02 ^de^	1.46 ± 0.11 ^a^
J	5.73 ± 0.11 ^g^	34.75 ± 0.92 ^bc^	1.41 ± 0.03 ^g^	5.96 ± 0.01 ^g^	1.00 ± 0.08 ^de^
K	6.86 ± 0.04 ^cd^	30.20 ± 1.59 ^ef^	1.66 ± 0.04 ^ef^	6.16 ± 0.06 ^b^	1.19 ± 0.14 ^bcd^
L	6.15 ± 0.17 ^f^	35.28 ± 1.18 ^bc^	3.78 ± 0.05 ^a^	6.12 ± 0.03 ^cde^	1.03 ± 0.04 ^de^
M	5.46 ± 0.10 ^gh^	39.12 ± 2.17 ^a^	0.67 ± 0.07 ^i^	6.15 ± 0.04 ^bc^	1.3 ± 0.13 ^abc^
N	6.59 ± 0.13 ^de^	40.16 ± 1.82 ^a^	1.13 ± 0.16 ^h^	6.05 ± 0.02 ^f^	1.3 ± 0.03 ^abc^
Mean	6.42 ± 0.97	34.42 ± 3.47	1.85 ± 0.89	6.13 ± 0.09	1.15 ± 0.20

Different letters indicate significant differences at *p* < 0.05. All the values (mean ± SD, *n* = 3) are expressed on a dry weight basis.

**Table 4:** Sugar and acrylamide analysis in 14 original-cut potato chip brands.

Brand	Glucose (mg/g)	Fructose (mg/g)	Sucrose (mg/g)	Acrylamide (μg/kg)
A	not detected	3.5 ± 0.73 ^d^	2.31 ± 0.18 ^gh^	168.43 ± 11.15 ^h^
B	2.5 ± 0.06 ^c^	2.47 ± 0.09 ^ef^	3.33 ± 0.22 ^f^	249.43 ± 8.98 ^g^
C	not detected	0.78 ± 0.02 ^i^	1.28 ± 0.15 ^i^	407.13 ± 24.55 ^e^
D	not detected	6.9 ± 1.03 ^a^	5.01 ± 0.32 ^d^	408.43 ± 8.05 ^e^
E	not detected	0.93 ± 0.06 ^hi^	2.3 ± 0.27 ^gh^	398.63 ± 11.3 ^e^
F	17.56 ± 0.8 ^a^	2.61 ± 0.27 ^e^	5.52 ± 0.2 ^c^	166.7 ± 9.6 ^h^
G	2.94 ± 0.55 ^b^	5 ± 0.15 ^b^	8.78 ± 0.79 ^a^	908.75 ± 53.5 ^b^
H	1.36 ± 0.05 ^d^	4.12 ± 0.4 ^c^	1.87 ± 0.17 ^h^	417.23 ± 18.1 ^e^
I	not detected	1.86 ± 0.37 ^g^	2.73 ± 0.13 ^g^	303.83 ± 10.58 ^f^
J	not detected	0.87 ± 0.04 ^hi^	3.33 ± 0.16 ^f^	231.73 ± 11.08 ^g^
K	0.75 ± 0.2 ^f^	1.35 ± 0.24 ^h^	4.78 ± 0.57 ^d^	464.65 ± 34.68 ^d^
L	0.98 ± 0.06 ^ef^	0.93 ± 0.1 ^hi^	4.7 ± 0.64 ^d^	127.05 ± 5.25 ^h^
M	not detected	2.02 ± 0.16 ^fg^	6.27 ± 0.33 ^b^	1101.78 ± 22.55 ^a^
N	1.32 ± 0.05 ^de^	1.95 ± 0.09 ^g^	4.2 ± 0.05 ^e^	671.73 ± 23 ^c^
Mean	1.96 ± 4.62	2.52 ± 1.84	4.03 ± 1.98	430.39 ± 283.29

Different letters indicate significant differences at *p* < 0.05. All the values (mean ± SD, *n* = 3) are expressed on a dry weight basis.

**Table 5:** Textural property analysis in 14 original-cut potato chip brands.

Brand	Hardness (gf)	Fracturability (gf)
A	670.42 ± 41.94 ^e^	167.5 ± 24.74 ^e^
B	540.72 ± 33.62 ^fg^	259.64 ± 34.91 ^e^
C	678.44 ± 22.38 ^e^	540.78 ± 33.68 ^c^
D	469.03 ± 24.21 ^g^	438.54 ± 23.54 ^d^
E	1103.6 ± 22.03 ^a^	857.77 ± 58.46 ^a^
F	1016.01 ± 50.14 ^b^	456.63 ± 28.67 ^cd^
G	771.93 ± 18.38 ^cd^	548.54 ± 42.35 ^c^
H	717.27 ± 33.6 ^de^	667.93 ± 81.5 ^b^
I	660.88 ± 59.47 ^e^	660.88 ± 59.47 ^b^
J	379.38 ± 20.92 ^h^	378.59 ± 21.97 ^d^
K	832.26 ± 19.14 ^c^	398.59 ± 68.11 ^d^
L	584.76 ± 39.41 ^f^	233.99 ± 22.31 ^e^
M	724.62 ± 16.6 ^de^	724.62 ± 16.6 ^b^
N	839.4 ± 55.42 ^c^	201.48 ± 70.6 ^e^
Mean	713.48 ± 203.18	466.82 ± 218.30

Different letters indicate significant differences at *p* < 0.05. All the values are indicated as mean ± SD (*n* = 3).

## Data Availability

The original contributions presented in this study are included in the article/Supplementary Materials. Further inquiries can be directed to the corresponding author.

## Supplementary Materials

The following supporting information can be downloaded at https://www.mdpi.com/article/10.3390/foods13244158/s1, Table S1: Origin and ingredient information of 14 original-cut potato chip brands from the Chinese Market. Table S2. Nutritional Composition and Percent Daily Values of 14 original-cut potato chip brands from the Chinese market.
