# Supplementary material for: Comprehensive Analysis of Physicochemical Properties and Sensory Attributes of Original-Cut Potato Chips in the Chinese Market
Source: Foods. 2024 Dec 22;13(24):4158. doi: 10.3390/foods13244158 (PMC11675445; doi:10.3390/foods13244158)
Supplement: Supplementary file 1 [file foods-13-04158-s001.zip › foods-3343787-supplementary.pdf]

Table S1 Origin and ingredients information of 14 original-cut potato chip brands from the Chinese market.

Brand	Place of Origin	Ingredients List
A	Hubei, China	Potatoes, vegetable oil, classic original flavoring (containing 5'-disodium ribonucleotides), TBHQ
B	Fujian, China	Potatoes, refined vegetable oil, potato-flavored seasoning (maltodextrin, sodium chloride, potato snowflake powder, monosodium glutamate, food-grade flavoring (containing dairy components), spices, silicon dioxide, aspartame (contains phenylalanine))
C	Guangdong, China	Potatoes, vegetable oil, sea salt seasoning (sodium chloride, monosodium glutamate, maltodextrin, 5'-disodium ribonucleotides, silicon dioxide)
D	Yunnan, China	Fresh potatoes, palm oil, pure original compound seasoning (sodium chloride, maltodextrin, monosodium glutamate, sucrose)
E	Guizhou, China	Potatoes, vegetable oil, sodium chloride, Sichuan pepper powder, monosodium glutamate, food additive (ethyl maltol)
F	Yunnan, China	Potato, vegetable oil, original seasoning (sucrose, sodium chloride, monosodium glutamate, dextrose, processed cheese, maltodextrin, yeast extract, spices, food additives (food-grade flavoring, silicon dioxide); certain raw materials (spices) in this product have undergone irradiation sterilization)
G	Yunnan, China	Potatoes, palm oil, original compound seasoning (sucrose, sodium chloride, monosodium glutamate, food-grade flavoring, compound soy sauce seasoning powder, onion powder, garlic powder, spices, silicon dioxide, aspartame (contains phenylalanine))
H	Tianjin, China	Potatoes, vegetable oil, sodium chloride, monosodium glutamate, paprika, 5'-disodium ribonucleotides, parsley flakes
I	Yunnan, China	Potatoes, vegetable oil, sodium chloride, monosodium glutamate
J	Guangdong, China	Potatoes, vegetable oil, sea salt seasoning (sodium chloride, monosodium glutamate, 5'-disodium ribonucleotides, silicon dioxide)
K	Hong Kong, China	Potatoes, vegetable oil, original seasoning, sucrose, sodium chloride
L	Shandong, China	Potatoes, vegetable oil, original seasoning powder (sucrose, maltodextrin, sodium chloride, monosodium glutamate, silicon dioxide, 5'-disodium ribonucleotides)
M	Yunnan, China	Potatoes, palm oil, original seasoning (sucrose, sodium chloride, monosodium glutamate, food-grade flavoring, compound soy sauce seasoning powder, onion powder, garlic powder, spices, silicon dioxide, aspartame (contains phenylalanine))
N	Liaoning, China	Potatoes, vegetable oil, original compound seasoning (containing flavoring)

Table S2. Nutritional Composition and Percent Daily Values of 14 original-cut potato chip brands from the Chinese market.

Brand	Serving size (g)	Energy kJ (% DV)	Protein g (% DV)	Total Fat g (% DV)	Carbohydrates g (% DV)	Sodium mg (% DV)
A	30	686 (8)	1.7 (3)	10.5 (18)	15.8 (5)	170 (9)
B	100	2257 (27)	5.2 (9)	34.0 (57)	51.6 (17)	540 (27)
C	60	1370 (16)	3.6 (6)	21.0 (35)	32.3 (10)	215 (11)
D	95	2131 (25)	5.2 (9)	33.3 (56)	47.7 (16)	318 (16)
E	100	2434 (29)	4.9 (8)	41.9 (70)	47.1 (16)	270 (14)
F	30	737 (9)	1.7 (3)	12.6 (21)	14.2 (5)	82 (4)
G	30	727 (9)	1.8 (3)	11.7 (20)	15.5 (5)	103 (5)
H	100	2287 (27)	5.5 (9)	34.3 (57)	54.4 (18)	660 (33)
I	100	2412 (29)	7.5 (13)	43.5 (73)	39.7 (13)	214 (11)
J	50	1171 (14)	1.9 (3)	18.0 (30)	27.8 (9)	231 (12)
K	30	681 (8)	1.9 (3)	10.5 (18)	14.4 (5)	201 (10)
L	45	1075 (13)	2.5 (4)	17.3 (29)	23.1 (8)	153 (8)
M	100	2312 (28)	6.0 (10)	34.0 (57)	56.0 (19)	224 (11)
N	100	2453 (29)	5.6 (9)	42.0 (70)	47.2 (16)	274 (14)

DV - Daily values
